# *Notes from the Field:* Drug Overdose Deaths in Hotels and Motels — United States, 2022–2024

**DOI:** 10.15585/mmwr.mm7524a2

**Published:** 2026-06-25

**Authors:** Andrea Stewart, Amanda Dinwiddie, April Wisdom, Thomas Gilson

**Affiliations:** ^1^Division of Overdose Prevention, National Center for Injury Prevention and Control, CDC; ^2^Cuyahoga County Medical Examiner’s Office, Cleveland, Ohio.

**Summary**

**What is already known about this topic?**

In 2024, at least 2,327 drug overdose deaths that were unintentional or of undetermined intent occurred in hotels and motels. Location-specific overdose data might help guide distribution of overdose prevention resources.

**What is added by this report?**

During 2022–2024, at least 9,651 drug overdose deaths occurred in U.S. hotels and motels; more than one half (56.2%) occurred in the same county where the decedent lived. A potential bystander (a person who was physically nearby and had an opportunity to intervene) was present for 34.7% of deaths, but a timely response was delayed or did not occur in 63.5% of these deaths. Substance use by the bystander was a reason for 29.7% of nonresponses or delayed responses.

**What are the implications for public health practice?**

Enhancing drug overdose prevention strategies in hotels and motels, including providing information about local treatment options and opioid overdose reversal medication distribution and education, might help prevent overdose deaths in these settings.

In 2024, at least 2,327 drug overdose deaths occurred in hotels and motels, the second most common location after houses and apartments (*1*). Analyses of overdose locations can help jurisdictions make decisions regarding optimal distribution of limited local resources for prevention, including opioid overdose reversal medications such as naloxone, and identify local options for substance use disorder treatment to prevent overdose deaths among residents (*2*–*4*). However, analyses of overdose locations do not always explicitly include hotels and motels (*4*). Public health interventions can benefit from improved understanding of the circumstances surrounding drug overdose deaths in hotels and motels, including whether local resources to prevent overdoses among residents might be used in these locations. CDC analyzed data from its State Unintentional Drug Overdose Response System (SUDORS) to characterize drug overdose deaths that occurred in hotels and motels during 2022–2024.

## Investigation and Outcomes

### Data Source and Analysis

SUDORS collects data on overdose deaths that were unintentional or of undetermined intent from death certificates, postmortem toxicology reports, and reports with contextual data about the circumstances of the death (e.g., coroner or medical examiner investigation reports, emergency medical services records, and medical records). CDC conducted a descriptive analysis of 2022–2024 SUDORS data from 47 jurisdictions (46 states and the District of Columbia).* The objective of this analysis was to describe certain characteristics of overdose deaths that occur in hotels and motels, including the demographic characteristics of decedents and the percentage who were residents of the same county where the hotel or motel was located, with the goal of identifying potential missed opportunities for intervention, prevention, and connection to treatment. This activity was reviewed by CDC, deemed not research, and conducted consistent with applicable federal law and CDC policy.^†^

### Decedent Characteristics, Bystander Responses, Opioid Involvement, and Naloxone Use

**Decedent characteristics.** Among 191,575 overdose deaths during 2022–2024 across 47 SUDORS-participating jurisdictions, 9,651 (5.0%) occurred in hotels and motels.^§^ Among the decedents, 71.3% were male, 74.6% were aged 25–54 years, and 64.6% were White (Table).^¶^ Among the 9,344 (96.8%) overdose deaths in hotels and motels for whom the decedent’s county of residence was known, 5,253 (56.2%) occurred in the county where the decedent lived.

**TABLE:** Number and percentage of drug overdose deaths in hotels and motels,* by demographic characteristics of decedents, types of drugs involved, bystander responses, and naloxone use — State Unintentional Drug Overdose Reporting System, United States,^†^ 2022–2024

Characteristic	No. (%)
**Total**	**9,651 (100.0)**
**Age group, yrs^§^**	
<15	16 (0.2)
15–24	391 (4.1)
25–34	2,115 (21.9)
35–44	2,907 (30.1)
45–54	2,182 (22.6)
55–64	1,617 (16.8)
≥65	422 (4.4)
**Sex^§^**	
Female	2,774 (28.7)
Male	6,877 (71.3)
**Race and ethnicity^§,¶^**	
American Indian or Alaska Native	186 (1.9)
Asian	74 (0.8)
Black or African American	2,042 (21.2)
Hispanic or Latino	890 (9.2)
Native Hawaiian or Pacific Islander	7 (0.1)
White	6,229 (64.6)
Other race or multiple races	130 (1.3)
Decedent lived in county of overdose**	5,253 (56.2)
**Presence and reasons for nonresponse of potential bystanders**	
Potential bystander present^††^	3,344 (34.7)
Did not respond or had a delayed response^§§^	2,122 (63.5)
Was spatially separated	682 (32.1)
Was using substances or alcohol	631 (29.7)
Did not recognize symptoms as an overdose	401 (18.9)
Did not recognize abnormalities	356 (16.8)
Was unaware decedent was using substances	279 (13.1)
Occurred in a public space; strangers did not intervene	45 (2.1)
**Drugs involved^¶¶^**	
Any opioid***	8,254 (85.5)
Illegally manufactured fentanyls	7,831 (81.1)
Any stimulant	6,662 (69.0)
Any opioid and any stimulant	5,450 (56.5)
**Naloxone administered^†††^**	**1,705 (20.7)**
By professional responder^§§§,¶¶¶^	913 (53.5)
By lay person^§§§^	373 (21.9)
By hotel staff member^§§§,^****	24 (1.4)
By unknown person^§§§^	530 (31.1)

* Data on place of residence, place of injury, and place of death were obtained from the death certificate. If the place of injury was missing or unknown, the place of death was used to determine whether the overdose death occurred in a hotel or motel.

^†^ Restricted to deaths with contextual data from jurisdictions with death certificates and contextual data for ≥75% of deaths for at least one 6-month reporting period during 2022–2024 in the following 47 jurisdictions: Alabama, Alaska, Arizona, Arkansas, Colorado, Connecticut, Delaware, District of Columbia, Florida, Georgia, Hawaii, Illinois, Indiana, Iowa, Kansas, Kentucky, Louisiana, Maine, Maryland, Massachusetts, Michigan, Minnesota, Mississippi, Missouri, Montana, Nebraska, Nevada, New Hampshire, New Jersey, New Mexico, New York, North Carolina, Ohio, Oklahoma, Oregon, Pennsylvania, Rhode Island, South Carolina, South Dakota, Tennessee, Utah, Vermont, Virginia, Washington, West Virginia, Wisconsin, and Wyoming.

^§^ Missing values were excluded from calculations of percentages. Percentages might not sum to 100 because of rounding. Fewer than 1% of deaths were missing age data and <5% of deaths were missing race and ethnicity classification data.

^¶^ Persons of Hispanic or Latino (Hispanic) ethnicity, regardless of race, were classified as Hispanic. Persons who were non-Hispanic were reported by their indicated single-race classification (American Indian or Alaska Native, Black or African American, White, or other race or multiple races).

** If county of injury was missing from the death certificate, county of death was used rather than county of injury to determine whether a decedent was a resident of the same jurisdiction. Denominator used in calculation of percentage was the total number of deaths with residence and place of injury or death (9,344).

^††^ Denominator was used to calculate the percentage of deaths without a response or with a delayed response. Bystander responses were not mutually exclusive. A potential bystander was defined as a person aged ≥11 years who was physically nearby either during or shortly before the drug overdose and potentially had an opportunity to intervene in or respond to the overdose. This did not include persons in different self-contained parts of larger buildings (e.g., a person in the same hotel but in a different hotel room) but did include persons in a different room nearby where they might have had the opportunity to intervene if they had known the decedent was using drugs (e.g., the bathroom).

^§§^ Denominator was used to calculate the percentage of deaths with potential reasons for an absence or delay in bystander response.

^¶¶^ A drug was considered involved in the death if it was listed as a cause of death on the death certificate or in the medical examiner or coroner report. Percentages might sum to >100 because drug categories were not mutually exclusive.

*** Deaths that had at least one opioid listed as a cause of death (i.e., illegally manufactured fentanyls, heroin, prescription opioids, and any other opioids) were considered opioid-involved deaths.

^†††^ Denominator used in calculating percentage was the total number of deaths with opioids involved.

^§§§^ Denominator used in calculating percentage was the total number of deaths with opioids involved with evidence naloxone was administered.

^¶¶¶^ Professional responder categories included emergency medical services responders and firefighters, law enforcement officers, and hospital or other health care staff members.

**** Hotel or motel employees were identified based on review of a write-in field in the data entry system.

**Bystander responses.** Potential bystanders** were present or nearby at the time of the fatal overdose for 3,344 (34.7%) of these overdoses in hotels and motels. Among these fatal overdoses, 2,122 (63.5%) had either no documented bystander response or a delayed bystander response. In approximately one third (631; 29.7%) of fatal overdoses for which a bystander was present but the response was delayed or did not occur, the bystander’s use of substances or alcohol was noted as a potential reason for the delayed or absent response.

**Opioid involvement and naloxone administration.** Opioids were involved in 8,254 (85.5%) overdose deaths in hotels and motels. In approximately one fifth (1,705; 20.7%) of these fatal overdoses, naloxone was administered. In at least 24 deaths, naloxone was administered by a hotel or motel employee.

## Preliminary Conclusions and Actions

Among overdose deaths that were unintentional or of undetermined intent that occurred in hotels and motels during 2022–2024, multiple potential opportunities for prevention were identified. More than one half (56.2%) of overdose deaths in hotels and motels occurred in the same county where the decedent lived, indicating that focusing local overdose prevention resources in these locations might be appropriate. During the same period, among fatal overdoses for which potential bystanders did not respond or had a delayed response, substance use by the bystander was identified as a reason in approximately 30% of cases, which is three times the percentage among all overdose deaths (8.7%–9.8%) (*1*). This finding suggests that some persons might go to hotels and motels for the purpose of using drugs with others. Among overdose deaths that occurred in hotels and motels, the percentage that involved opioids (85.5%) was higher than that of all overdose deaths (73.4%–81.7%) (*1*). Timely administration of opioid overdose reversal medications by a bystander, such as a hotel or motel employee, can help prevent additional overdose deaths. 

These findings suggest that including hotels and motels in overdose surveillance data analyses and, depending on the local context and resources, partnering with hotel and motel operators when planning overdose prevention programs might help public health officials improve the safety of guests and help prevent overdose deaths in these locations. Evidence-based strategies might include providing staff members with training on how to administer opioid overdose reversal medications, as well as education on overdose recognition and Good Samaritan Laws (*5*). In addition, overdose reversal kits and information about local substance use disorder treatment options might be made available in common areas of hotels and motels.

## Footnotes

* Restricted to deaths with contextual data from jurisdictions with death certificates and contextual data for ≥75% of deaths for at least one 6-month reporting period during 2022–2024 in the following 47 jurisdictions: Alabama, Alaska, Arizona, Arkansas, Colorado, Connecticut, Delaware, District of Columbia, Florida, Georgia, Hawaii, Illinois, Indiana, Iowa, Kansas, Kentucky, Louisiana, Maine, Maryland, Massachusetts, Michigan, Minnesota, Mississippi, Missouri, Montana, Nebraska, Nevada, New Hampshire, New Jersey, New Mexico, New York, North Carolina, Ohio, Oklahoma, Oregon, Pennsylvania, Rhode Island, South Carolina, South Dakota, Tennessee, Utah, Vermont, Virginia, Washington, West Virginia, Wisconsin, and Wyoming.

† 45 C.F.R. part 46.102(l)(2), 21 C.F.R. part 56; 42 U.S.C. Sect. 241(d); 5 U.S.C. Sect. 552a; 44 U.S.C. Sect. 3501 et seq.

§ Place of residence, injury, and death were obtained from the death certificate. If the place of injury where the overdose occurred (e.g., a hotel or motel, school, or house) was missing or unknown, the place of death was used to determine whether the overdose death occurred in a hotel or motel. If the county of injury was missing from the death certificate, the county of death was used in place of county of injury to determine whether a decedent lived in the same jurisdiction.

¶ Persons of Hispanic or Latino (Hispanic) ethnicity, regardless of race, were classified as Hispanic. Persons who were non-Hispanic were reported by their indicated single-race classification.

** A potential bystander was defined as a person aged ≥11 years who was physically nearby either during or shortly before the drug overdose and potentially had an opportunity to intervene in or respond to the overdose. This did not include persons in different self-contained parts of larger buildings (e.g., a person in the same hotel but in a different hotel room) but did include persons in a different room nearby where they might have had the opportunity to intervene if they had known the decedent was using drugs (e.g., the bathroom).
